# “He is just Ken:” deconstructing hegemonic masculinity in Barbie (2023 Movie)

**DOI:** 10.3389/fsoc.2024.1320774

**Published:** 2024-04-05

**Authors:** Dikmen Yakalı

**Affiliations:** Department of General Culture Courses, Izmir Institute of Technology (IZTECH), Izmir, Türkiye

**Keywords:** Barbie, Ken, postfeminism, children's media, Barbie 2023 Movie, hegemonic masculinity, gender identity, hypermasculinity

## Abstract

Scholars have consistently explored Barbie in various contexts, often subjecting it to critical analysis. However, the release of the Barbie 2023 Movie has shifted our focus from Barbie to Ken, marking the first occasion when Barbie has provided a platform for exploring representations of masculinity both in the patriarchal society and in popular culture. This article aims to investigate how the 2023 Barbie movie deconstructs symbols of hegemonic and toxic masculinity and its performative aspects within the framework of (post)feminist discourse. It examines how the movie satirically employs symbols of traditional, hegemonic masculinity to challenge normative masculine ideals prevalent in our patriarchal society. The movie -through its popularity- significantly contributes to mainstream postfeminist media culture, creating a platform where discussions on masculinity, its associated crises, and the broader gender wars, along with their existential ramifications, become unavoidable. Exploring the ways masculinities are problematized and contested within postfeminist media culture, I argue that Ken, within this narrative, is positioned as the latest icon of postfeminist masculinity, symbolizing a critical juncture in the ongoing discourse on gender roles and identities.

## 1 Introduction

Scholars have consistently explored Barbie in various contexts, often subjecting it to critical analysis as a domain where ideas are scrutinized. However, the release of the Barbie 2023 Movie has shifted our focus from Barbie to Ken, marking the first occasion when Barbie has provided a platform for dissecting representations of masculinity in the postfeminist media landscape. This shift is evident right from the movie's poster, which boldly proclaims, “Barbie is everything. He is just Ken.”

This article aims to investigate how the 2023 “Barbie” movie which is directed and co-written by Greta Gerwig (and Noah Baumbach) employs symbols of hegemonic and toxic masculinity and its performative aspects within the framework of postfeminist discourse (Gerwig and Baumbach, 2023). It examines how the movie satirically employs symbols of hyper and toxic masculinities to challenge normative masculine ideals prevalent in a patriarchal society. This study suggests that the Barbie Movie is intricately connected to the postfeminist media culture, creating a platform for discussions about feminism, patriarchy, and the changing ideas about masculinity. Contemporary manifestations of feminism, often aligned with neoliberal values that prioritize individual empowerment over collective societal change, have become commodified and politically diluted within postfeminist media and celebrity culture (Gill and Scharff, 2011). This commodification is critiqued for transforming feminism into a fashionable yet superficial concept, devoid of its political commitment and transformative potential. Under this framework, the Barbie 2023 Movie emerges as a significant subject of analysis. The film not only commodifies feminism and the so-called gender wars but also leverages the extensive academic criticism that has historically targeted Barbie. However, my contention is that the movie -through its popularity- significantly contributes to mainstream media culture, creating a platform where discussions on masculinity, its associated crises, and the broader gender wars, along with their existential ramifications, become unavoidable. It sheds light on the ways masculinities are problematized and contested within postfeminist media culture. Therefore, I argue that Ken, within this narrative, is positioned as the latest icon of postfeminist masculinity, symbolizing a critical juncture in the ongoing discourse on gender roles and identities.

In the realm of Kendom, Barbie is perceived as the dominating force that Kens must contend with. In the Real World, while men are at times portrayed as antagonists to confront, the presence of a postfeminist masculinity embodied by Ken prompts us to contemplate how the film actually frames the patriarchal system as the antagonist in all the narratives it presents. Despite Barbies governing Barbieland, which operates as a matriarchal system, it fails to alter the underlying structures of the inherently biased system. When a hierarchical system persists, featuring varying degrees of power, marginalization, and everything in between, the system functions much as it always has. This constitutes a profound critique of the patriarchal system within the movie. The gender of those in power is not the core issue; the fundamental challenge lies in reshaping the system and discerning who the true adversary is. This enduring critique throughout the movie establishes it as a feminist text.

Through the analysis of the film, this article seeks to examine how specific phrases, signs, symbols and narratives are used in the dialogues or visuals as recurring themes and patterns to deconstruct social and cultural norms of masculinity. This approach allows me to not only identify themes such as humor, satire, or the use of specific symbols but also to explore how these elements actively participate in the construction and enactment of gender identities within the film. It enables a deeper understanding of how characters' actions, expressions, and interactions perform masculinity and how these performances challenge or subvert traditional norms. It's important to note that the film's transcriptions are available online as open-source material.

In conversations regarding the film's narrative and overall cinematic creation, I intentionally use the term “the film” without singling out the writer-director Greta Gerwig and co-writer Noah Baumbach. This choice is deliberate and should not be interpreted as an attempt to diminish or disregard the importance of their roles. I aim to emphasize and acknowledge that the existence of this film is also contingent upon the decisions and actions of Mattel and Warner Bros. executives who chose to produce and promote it. This approach serves to recognize the broader popular culture industry and Mattel's responsiveness to, and promotion of, its own critiques, demonstrating a clear intention to engage in this endeavor.

### 1.1 Barbie's global relevance

Barbie, the iconic doll created by Ruth Handler in 1959, has transcended its role as a toy to become a cultural phenomenon through animated films, TV shows and magazines. She became a symbol of beauty, fashion, and femininity. Across various contexts, Barbie has been a symbol of materialism, cultural adaptation, and globalization. She embodies ideals and values that transcend national boundaries, influencing perceptions of beauty, gender roles, and consumer culture worldwide. Her impact on societal norms and individual identity formation, especially among young girls, is significant, prompting both admiration and criticism. With an expansive array of merchandise that includes decorations, stationery, and clothing, it has become a part of our material culture and daily lives. The Barbie 2023 Movie, directed by Greta Gerwig, has significantly contributed to this legacy, becoming the biggest film of the year with a $1.45 billion in global box office earnings (Statista, 2024). This financial success is a testament to Barbie's enduring appeal and the effective marketing strategies employed by Mattel and its partners (Walfisz, 2023).

The craze for Barbie-themed merchandise around the movie's release further underscores the brand's global reach. The film's success and the associated merchandise boom are expected to boost global sales of Barbie dolls, which had seen a decline from record growth during the pandemic (Reid, 2023). From achieving record-breaking box office earnings to influencing fashion trends and consumer behavior, Barbie's reach is truly global. The 2023 movie and its aftermath not only reinforce Barbie's status as an icon of popular culture but also illustrate the brand's ability to evolve and remain relevant in the changing landscape of media and consumer preferences.

Over the years, Barbie has generated significant academic interest, leading to a multitude of scholarly works exploring its influence on children's development, gender roles, body image, and societal values (McDonough, 1999; Rakow and Rakow, 1999; Toffoletti, 2007) in the same brackets. The evolution of Barbie's critique within academic discourse has been marked by notable shifts, particularly in how academia perceives popular culture, as observed by scholars like Rogers (1999). Over time, Barbie has faced criticism for being portrayed as a symbol of objectified sexuality. This feminist lens positions Barbie as a mechanism that perpetuates and bolsters the male-dominated consumer culture (Steinberg, 1997, 2009; Varney, 2002, p. 155). She has been criticized for promoting impossibly slender and disproportionate body standards of emphasized femininity that are associated with numerous issues among teenage girls and young women (Urla and Swedlund, 1995; Varney, 2002).

On the opposite end of the spectrum, Barbie is seen as a feminist figure, offering liberating potential for young girls. Barbie embodies careers beyond motherhood, encouraging girls to envision diverse future possibilities (Brill, 1995). This perspective serves as the central premise on which Barbie 2023 is initially built, only to undergo a continuous process of deconstruction throughout the film. However, this view has also been challenged, with arguments asserting that playing with Barbie dolls did not necessarily lead little girls to believe that they could be anything (Sherman and Zurbriggen, 2014).

There exists an alternative viewpoint in relation to those who assert that “it is just a doll,” indicating that the inherent meaning is not contained within the doll but rather constructed externally by how it is played with (Reid-Walsh and Mitchell, 2000). In fact, it has been observed that girls who play with Barbie can, and often do, engage in games that challenge normative ideals (Brill, 1995; Rand, 1995; Reid-Walsh and Mitchell, 2000).

The producers of Barbie are attuned to critical discourses surrounding the doll and actively position their product in response. For example, responding to the concerns and critiques of Barbie's bodily standards, Mattel initiated innovative product lines: “Barbie Fashionista” and “M2M—Made to Move.” These initiatives involved the creation of dolls designed to embrace a diverse range of body images which also appear in the movie. However, studies subsequent to M2M investigated how exaggerated features, such as those seen in Barbie and Ken dolls, can influence expectations and perceptions of weight (Saccone and Chouinard, 2019) or how levels of body appreciation are influenced by Barbie play in comparison to Lego Friends play sets (Webb et al., 2023).

In my other studies, I have contended that Barbie has transitioned into a postfeminine icon and, concurrently, a postfeminist figure. This status is attributed to her unique ability to navigate and harmonize seemingly conflicting historical notions of femininity, bridging the gap between seemingly incongruent feminine and feminist subject positions. This positioning can be seen as occupying an intermediary space. I argued that this postfeminine identity of Barbie is a narrative identity that is dialogically constructed, reflecting the influence of poststructuralist feminist and queer theories (Yakalı-Çamoglu, 2020a,b). However, in this latest movie, her identity is reset once again, and she asserts herself as the ultimate “feminist” subject. She is determined to resist any meanings or ideas imposed on her, aspiring instead to be an “ordinary” Barbie/woman. This new stance allows her to create and construct meanings and ideas, rather than being merely the subject or object of conversation, narrative, or discourse (Yakalı, 2024). With this new movie, on the other hand, Barbie brings masculinity into the spotlight as well. Ken doll who was named after Ruth Handler's son, and made his debut in stores in 1961, had lived in the shadows until this 2023 movie (Carlin, 2023). By highlighting the dialogical construction of Ken's identity and exploring its subversive potential, this study contributes to a deeper understanding of the interplay between masculinity, feminism, and postfeminism in the context of Barbie's cultural influence.

### 1.2 Theoretical framework: postfeminist masculinities

Barbie 2023 Movie positions itself within ongoing theoretical and academic discussions related to gender and identity through a (post)feminist deconstructive stance. It also highlights the performative nature of gender, emphasizing that gender has no intrinsic qualities but is instead constructed through performances (Butler, 1988). The satirical and humorous style it employs in discussions on gender wars, critiques of patriarchy, and the state of gender identities in this postfeminist landscape opens a space for discussing various aspects of gendered lives.

The concept of postfeminism remains highly debated (see, Lotz, 2001, p. 11–113). It may denote a sense of “after” in relation to feminism, but it can also signify resistance or rejection of feminism itself (Genz and Brabon, 2009, p. 3–4). Postfeminism indicates the transformation and infusion of feminist discourse and categories into media and popular culture products (Lotz, 2001; Genz, 2009; Genz and Brabon, 2009). It also represents a cultural sensibility emerging from and reacting to feminism's legacy. According to McRobbie (2004, 2008) and Gill (2007, 2014), postfeminism is not a unified ideology but consists of conflicting discourses on gender roles. This sensibility is characterized by an emphasis on individualism, empowerment, and self-regulation, intertwined with the broader neoliberal context. Postfeminism acknowledges feminist achievements but simultaneously implies their redundancy in the contemporary era, often trivializing ongoing feminist struggles. Negra's (2009) analysis illustrates how postfeminist media celebrates female achievements in male-dominated spheres while subtly undermining feminist politics.

Gill's (2007) approach, which emphasizes the study of postfeminist media culture and necessitates a shift from relying on a fixed, authentic feminism to drawing from postmodern and constructivist perspectives for examining gender articulations, will be relevant for the purposes of this study. According to Gill (2007, p. 254) twenty-first-century media consistently highlights certain themes and structures in the representation of gender, including the embodiment of femininity; a transition from objectification to subjectification; emphasis on self-surveillance, discipline, and control; individualism; the power of choice; a paradigm of reinvention; the interplay and intertwining of feminist and anti-feminist ideas; the sexualization of culture; consumerism; and the commodification of differences. While Gill primarily discusses these themes in the context of femininity, they also offer a crucial framework for examining masculinities in postfeminist contexts. For instance, the portrayal of masculinity through both feminine and homosexual gazes suggests a transition from subjectification to objectification. Consequently, there is an emphasis on self-surveillance, discipline, and control which can be explored in how masculinity is represented and negotiated in media. The power of choice and the paradigm of reinvention may reflect the contemporary man's wavering between traditional and new masculinities, challenging and reshaping the boundaries of what it means to be masculine in a postfeminist era. Therefore, employing Gill's framework to analyze these themes in the representation of masculinities can uncover the ways in which gender is constructed, performed, and contested in postfeminist media culture.

The so-called crisis in masculinity dates to the last century, where male identity is depicted as fractured, vulnerable, and constrained. In a patriarchal society, men are conditioned to be rational and aggressive, neglecting their emotional and experiential life. Many stereotypes lead to the entrapment of men within these very stereotypes, with machismo emerging as self-destructive and masochistic (Horrocks, 1994; Kimmel, 2017). Definitions of manliness have evolved in response to feminism, and the crisis in masculinity has set the stage for the emergence of hypermasculinity and toxic masculinity (Kimmel, 1996). Hypermasculinity refers to an exaggerated or extreme form of traditional masculinity found in a heteronormative patriarchal society which emphasize traits like physical strength, aggression, dominance, emotional suppression, and the devaluation of characteristics and behaviors perceived as feminine (Vokey et al., 2013). It often suggests a firm belief in male superiority and performances of stereotypical male gender roles associated with power, dominance and control which contribute to issues such as sexism, misogyny, and violence against women (Alonzo and Guerrero, 2009). Incels, or involuntary celibates, also exemplify the resistance to postfeminist culture, often exhibiting extreme forms of toxic masculinity, including misogyny and violence, in reaction to their perceived marginalization in romantic relationships (Stijelja and Mishara, 2022; Bogetić et al., 2023). This resistance underscores a clash between evolving gender norms and deeply rooted patriarchal attitudes.

Gill (2016, 2017) suggests that postfeminism should continue to be used as a critical tool to understand the coexistence of feminist and anti-feminist ideas within media culture. This study positions postfeminism as a critical framework for unpacking the interaction between feminist and anti-feminist narratives in media culture to examine masculinities. The postfeminist media and cultural landscape is characterized by a significant tension between, on one hand, traditional, heteronormative masculinities that valorise physical strength, dominance, and emotional restraint, including its more problematic forms like hypermasculinity and toxic masculinity, and on the other hand, the celebration of alternative masculinities. This phenomenon can also be interpreted as a “double entanglement,” applicable to the representations of masculinities in postfeminist media culture. It refers to the simultaneous incorporation and undermining of feminist achievements within the media, thereby creating a complex web of both progressive and regressive narratives surrounding masculinities (McRobbie, 2008). The alternative masculinities advocate for emotional intelligence, empathy, and the dissolution of the binary between strength and vulnerability, encapsulating what Gill (2014) identifies as “unheroic” masculinities.

Postfeminist media culture has significantly reshaped the representation of masculinities. Tasker and Negra (2007, p. 21) introduced the concept of postfeminist masculinity as a discourse that celebrates the strength of women while offering subtle critiques or gentle mockery of stereotypical masculinity. In simpler terms, postfeminist masculinity portrays stereotypical masculinity as foolish or comical, and at times, even portrays it as immature or inadequate, with the intent of emphasizing the capabilities and independence of women (Macaluso, 2018). Recurring depiction of men as somewhat hapless or inept “victims” or “losers” within the context of the “sex wars,” all the while presenting feminism as extreme, outdated, and, in some cases, redundant or unnecessary also becomes a part of postfeminism (Gill, 2014, p. 191). Ken's “blonde fragility” in the movie also refers to the concept of the New Man in the 1980's, which presented women with “the possibility of an active female gaze” (Cohan, 2007, p. 182). Gill (2014) exploration of “unheroic masculinity” in popular fiction reveals a departure from traditional portrayals of male characters. Instead of embodying flawless heroism, these characters display vulnerabilities and flaws, reflecting a broader critique of traditional male dominance. This portrayal aligns with Connell and Messerschmidt's (2005) expanded concept of hegemonic masculinity, which now includes subordinated masculinities that challenge the dominant forms (Connell, 1995). In postfeminist narratives, men are often shown as not always strong or in control, a stark contrast to older ideas of manhood, suggesting a redefinition of what it means to be a man in contemporary society.

Another genre within this landscape is lad flick films which often humorously depict the juvenile nature of traditional masculine values and ideals as the product of an anxiety-ridden pursuit of collective male approval (Nixon, 2001). The comedic tension in these films often stems from the male protagonist's struggle to live up to or maintain unrealistic versions of masculinity, as dictated by their male peer group (Gill and Hansen-Miller, 2011, p. 39). The concept that manhood is homosocial—that is, men need to prove themselves to each other rather than to women—becomes the main theme to be deconstructed (Kimmel, 1996). Eventually, the “lad” character is compelled to grow up and overcome their subordination to homosocial values to become a proper adult.

The narratives and character developments within lad flick may reflect elements of “hybrid masculinities.” Hybrid masculinities refers to a conceptual framework within the field of gender studies that examines how contemporary masculinities incorporate elements traditionally considered feminine or otherwise not aligned with hegemonic masculinity (Bridges and Pascoe, 2014). This approach suggests that men's identities are increasingly becoming a blend of traditional masculine norms and those characteristics or behaviors that have historically been marginalized or devalued in men.

Mocking hypermasculinity and toxic masculinity in movies serves as a critique within postfeminist media culture. This form of ridicule can subvert and question traditional narratives around masculinity, introducing ambivalence by blending humor with critique, thus reflecting the mixed sentiments of postfeminist media culture. It enhances consumer appeal by making serious critiques more accessible and entertaining, potentially normalizing alternative masculinities by presenting them as preferable to their hypermasculine and toxic counterparts. Ultimately, by engaging with feminist discourse and challenging problematic aspects of traditional masculinity, Barbie 2023 contributes to the ongoing dialogue about gender norms, balancing critique with commercial viability in a neoliberal context. While mocking hyper and toxic masculinity might seem progressive, it can also provoke backlash from those who feel their identities or values are being threatened (Dosser, 2022).

It can be argued that postfeminist media landscape is an arena for the so-called “gender wars” which aptly describes the ongoing conflicts and debates in postfeminist media culture surrounding gender roles and identities. These wars are characterized by a reevaluation of traditional gender roles, a backlash against feminism, and contradictory representations of empowerment. The struggle for gender equality, negotiation of masculinities, and the role of intersectionality in these debates further compound the complexity of these wars (Gill and Donaghue, 2013). Digital and social media have amplified these conflicts, providing platforms for a multitude of voices and perspectives, sometimes leading to polarization (Kolehmainen, 2012). The Barbie Movie becomes the ultimate postfeminist icon of our media landscape by making these gender wars the central theme of a film that has reached a wide and diverse audience. It cleverly turns its critiques into a major theme, generating billions in revenue, and counterattacks by positioning Barbie as a “feminist” character who will liberate women from their misery.

However, it must be noted that the Barbie 2023 Movie is also a feminist text, as it suggests the real enemy in gender wars is the patriarchal system rather than gender itself, representing a significant and progressive narrative shift within postfeminist media culture. This approach aligns it more closely with feminist critiques of societal structures, moving beyond individual behaviors, identities, or personal choices to address the systemic foundations of inequalities.

### 1.3 The emplotment of the movie

The movie begins with a scene reminiscent of Stanley Kubrick's “2001: A Space Odyssey” and continues to build upon the scholarly perspective that posited Barbie play as a means for young girls to envision lives beyond the roles of wives and mothers. Then, we go to Barbieland, a matriarchal society inhabited by various Barbie and Ken dolls, along with a group of discontinued dolls who face societal exclusion due to their unconventional traits. Barbie grapples with existential concerns after experiencing physical changes overnight, including bad breath, cellulite, and flat feet. Weird Barbie, an outcast who functions as the mentor character, informs Barbie that she must locate the child playing with her in the real world to cure these afflictions. Ken secretly joins her on this journey.

Their quest leads them to Venice Beach, LA, where Barbie realizes that it is not a matriarchal society and that the goals of feminism have not been attained. She stands up to a man who gropes her, and this results in a brief arrest. When this draws the attention of Mattel's CEO, the firm orders Barbie and Ken's capture. Barbie eventually finds her owner, Sasha, a teen girl who criticizes her for promoting unrealistic beauty standards and creating a backlash of feminism. Barbie's existential crisis mirrors that of Gloria, Sasha's mother, and a Mattel employee, who began playing with Sasha's old Barbie toys, unintentionally setting off Barbie's internal turmoil and existential crises. Mattel tries to put Barbie in a toy box for remanufacturing, but with the help of Gloria and Sasha, she escapes and returns to Barbieland, pursued by Mattel executives.

Ken returns to Barbieland after learning about patriarchy and shares his newfound knowledge with the other Kens. The Kens assume control, relegating the Barbies to submissive roles. Despite Barbie's efforts to revert to the previous order, her attempts fail, leading to her descent into depression. However, Gloria steps in and delivers an empowering speech that addresses the contradictory expectations placed on women in society, restoring Barbie's self-confidence. With the support of Sasha, Weird Barbie, and Allan, Barbie and Gloria rally the Barbies to break free from their subordination. They manipulate Kens into war, preventing them from establishing male dominance in Barbieland.

The Barbies ultimately reclaim their power, having personally experienced systemic oppression, and commit to rectifying the flaws in their previous society. They emphasize the importance of fair treatment for all, marking a significant shift in their approach to governance.

Barbie and Ken reconcile, acknowledging their mistakes. Ken struggles with his sense of purpose without Barbie, but she encourages him to discover an autonomous identity. Barbie, still uncertain about her own identity, encounters the spirit of Ruth Handler. Ruth explains that Barbie's story has no predetermined ending, and her evolving history transcends her origins. After bidding farewell to the Barbies, Kens, and Mattel executives, Barbie decides to become human and return to the real world as an “ordinary” woman.

## 2 Results

### 2.1 Structure of feeling and insecurity

Barbie 2023 Movie constructs a postfeminist story universe in Barbieland. Postfeminist masculinity serves as an analytical lens for understanding masculinity in the specific context of this movie, where multiple aspects coexist simultaneously. The film suggests that despite significant progress toward gender equality, achieving some feminist goals, the overarching patriarchal system remains, continuing to adversely affect people of all genders. This perspective prompts a reevaluation of traditional gender roles and also shifts attention toward issues related to men and masculinity.

One aspect of postfeminist masculinity explored in the film is its invitation for us to reflect on the current state of masculinity. A primary question it raises is to what extent does this hybrid masculinity incorporate emotional expression? In line with lad flick genre or unheroic masculinity of the postfeminist media landscape, the film challenges traditional norms that discourage men from openly expressing emotions or vulnerability. Ken who is portrayed as childish and insecure challenges the conceptualization of “heroic” men of the patriarchal narratives. It advocates the idea that men possess feelings and should have the freedom to articulate a full spectrum of emotions.

The initial impression we gather of Ken revolves around his deep-seated insecurity and his desire to make a favorable impression on Barbie. This sense of insecurity within the context of Barbieland is explicitly articulated by the narrator in the very first scene that introduces the stereotypical Ken, as well as the other Kens, on the beach:

“Barbie has a great day every day, but Ken only has a great day if Barbie looks at him.” (00:08:04)

In this scene, Ken injures himself while attempting to impress Barbie by confronting the plastic waves. Shortly after, we observe him engaging in a juvenile competition with the Asian Ken, displaying readiness for a potential fight. However, Ken also remarks that he would “beach him off” if he weren't severely injured, indicating an inclination for aggression. The interaction between Ken and the Asian Ken encapsulates the themes of hypermasculinity. These behaviors highlight the struggle to adhere to exaggerated masculine ideals, fostering insecurity about gender performance within their homosocial group. This scenario resonates with Kimmel's (1996) discussion on the crisis in masculinity, where male identity is depicted as fractured and constrained by patriarchal expectations. Ken also resonates with the “lad flick” character who feels compelled to showcase his prowess both within his homosocial group and to the Barbies. The Asian Ken, in response, appears to belittle him, questioning why he's displaying such emotion. This interaction serves as a reminder that both the expression of emotions and resorting to physical conflict driven by emotions are viewed critically in our postfeminist society. Consequently, throughout these scenes, we witness the characters grappling with their identities and feeling insecure about their gender performance. This portrayal aligns with the notion of “hybrid masculinities,” where traditional and non-traditional masculine behaviors coexist and often conflict (Bridges and Pascoe, 2014).

As they make their way to the hospital van, Ken is overcome with desperation and clings to Barbie:

“Ken- Barbie, hold my hand!

Barbie- You're okay.

“Ken- Stay with me, Barbie!” (00:10:25)

The doctor Barbie examines the X-ray film and confirms that there is no fracture, reassuring Ken that he will be okay. In response, Ken experiences a blend of relief and remorse for his previous actions, and he replies:

“Shredding waves is much more dangerous than people realize.” 00:10:32

Barbie answers with an unemotional tone:

“You're very brave, Ken.”

Ken's reliance on Barbie's attention for validation and his subsequent expressions of vulnerability and desperation encapsulate the “double entanglement” of postfeminist media culture (McRobbie, 2008). Ken's actions, juxtaposed with Barbie's unemotional responses and insincere praise, critique the traditional gender dynamics perpetuated by patriarchal society. Barbie's treatment of Ken, particularly in praising his “beaching,” deconstructs the societal dynamic where women often bolster the egos of men, a dynamic rooted in hegemonic masculinity (Tannen, 1992; Walker, 2020).

### 2.2 Ken's existential crises

Ken undergoes an existential crisis, reflecting the broader challenges faced by masculinity in the postfeminist era. He experiences two distinct existential crises in the Movie. First, in Barbieland, he grapples with a profound sense of identity loss. In this fantastical world, he lacks agency, power, occupation, and even a place to call home. He often serves as a mere sidekick or helper during beach outings or parties. This mirrors the way women have historically been positioned in a typical patriarchal society, often relegated to secondary roles, or rendered invisible in the male-dominated world. This also aligns with the dynamics of girls' play, where male figures frequently assume secondary roles.

The film's exploration of Ken's identity crisis and Barbie's encouragement for him to find self-definition beyond their relationship directly engages with feminist critiques of traditional gender roles and the concept of individual agency. Barbie's response to Ken's existential dilemma is articulated in the words of Barbie:

“Maybe it's time to discover who Ken is... you have to figure out who you are without me. You're not your girlfriend. You're not your house, you're not your mink…. You're not even beach. Maybe all the things that you thought made you aren't... really you. Maybe it's Barbie and... it's Ken.” (01:35:25)

This echoes feminist calls for autonomy and self-realization that challenge patriarchal structures which often define individuals by their roles in relation to others.

Furthermore, the movie's subversion of traditional gender roles, as demonstrated through Ken's vulnerability and search for identity, aligns with postfeminist media culture's approach to gender representation. Postfeminism, with its contradictory relationship to feminism, both utilizes and critiques feminist gains by highlighting the limitations of traditional gender norms while exploring the complexities of identity in the contemporary era (McRobbie, 2004; Gill, 2007). For example when Ken says: “I just don't know who I am without you.” Barbie answers: “You're Ken.” He desperately goes on: “But it's ‘Barbie and Ken.' There is no just “Ken.” That's why I was created. I only exist within the warmth of your gaze.” This kind of a subjectivity without agency have been what feminism have challenged. Barbie's encouragement for Ken to redefine himself beyond societal expectations and the “warmth of [her] gaze” resonates with the postfeminist emphasis on individualism and self-regulation, albeit through a feminist lens that advocates for the dismantling of restrictive gender norms.

Thus, the binary points of identification; and the dichotomous ways of defining identities in a heteronormative relationship are challenged. The film suggests that these conventional binaries are being questioned or undermined in the context of gender and identity politics. The postfeminist subjectivities, which are closely connected to neoliberal consumerism under the auspices of choice and empowerment, also call for reconsideration.

Ken's second existential crisis occurs when he enters the Real World. Here, he suddenly becomes aware of the presence of patriarchy and the apparent dominance of men. However, he soon realizes that merely being a man does not automatically grant him a place in this world. To succeed, he needs education, financial resources, experience, and qualifications, much like women do. He also discovers that women hold various occupations, as evident in the doctor scene, and that similar rules apply to both genders. Therefore, in the postfeminist era, being a man in a patriarchal society is not as straightforward as it may seem.

As Ken experiences an overwhelming sense of happiness upon discovering that he has a place as a male in the Real World, he becomes eager to learn more about it. He decides to visit the library at Sasha's school and ends up stealing a few books. This act is a reference to the concept of bibliotherapy and reflects the postfeminist world's obsession with self-improvement through self-help books, courses, and therapies (Cohan, 2007). In a society where everyone is striving to find their place or narrative identity within an ever-evolving context, Ken selects four books. This act serves as a deconstruction of the self-help and makeover paradigm, with Ken choosing the books: “The Origins of the Patriarchy” by Godfrey Hogarth; “Why Men Rule (Literally)” by Richard Merritt and “Men and Wars” and a last one titled “Horses” by Ryan Bessin, all of which are fictitious books.

Subsequently, we witness his transformation of Barbieland into Kendom, effectively giving it an extreme makeover through the themes of these books. This time the film deconstructs patriarchy through this paradigm and identifies the symbols of hegemonic masculinity and deconstructs them.

### 2.3 Deconstructing hypermasculinity through symbols

As he is unable to belong in the Real World, Ken returns to Barbieland to enlighten the other Kens about the concept of patriarchy. Ken's transformation and the establishment of Kendom within Barbieland offer an illustration of the makeover paradigm, particularly in the context of gender roles and identities. The makeover paradigm, often associated with postfeminist media culture, typically emphasizes self-reinvention and transformation as a path to empowerment. However, Ken's humorous makeover and the subsequent establishment of Kendom reverse this narrative, showcasing a transformation that reinforces patriarchal and hegemonic norms rather than challenging them. This twist also provides a critical commentary on the limitations and potential repercussions of the makeover paradigm.

Ken establishes Kendom by orchestrating the Barbies into submissive roles mirroring those observed in the Real World. Barbies obediently serve men beverages and food, offer foot massages, and willingly embrace their subordinate positions. Meanwhile, the Kens readily embrace and adopt any symbols and acts associated with hegemonic, hyper or toxic masculinity that Ken had observed in the Real World.

Ken's initial appearance in traditionally “feminine” colors such as pink, white, and turquoise and his later shift to “masculine” colors such as navy blue and black reflect a deeper narrative on the fluidity of gender identity and the performative nature of gender roles, as discussed by Butler (1990). Ken's appearance undergoes a significant transformation in the movie. In the Real World, he comes across a picture of Sylvester Stallone, inspiring his choice of wardrobe. This includes a fur coat, a headband, a revealing six-pack, and leather half-gloves that leave his fingers exposed. Moreover, he wears three wristwatches simultaneously, prompted by an incident where a woman asked him for the time, leading Ken to believe he's finally gaining respect and recognition in the Real World. He even layers two pairs of sunglasses atop each other, thinking it looks cool, though it renders him somewhat absurd. All the other Kens dress in cowboy costumes.

Ken transforms Barbie's dream house into what he calls “Ken's Dojo Mojo Casa House.” Parked outside is a robust, masculine van used by the rangers. The space is stocked with sports equipment, including American football gear, boxing gloves, golf equipment, and a mini fridge for storing beer, which they seem to consume incessantly alongside snacks. In every scene set in Kendom, a visible jar of protein powder used by bodybuilders is featured. The display of protein powder as a symbol of the artificial construction of hypermasculinity aligns with Butler's (1990) notion of gender performativity. The emphasis on Ken's six-pack abs as a result of this artificial enhancement further illustrates the performative aspects of masculinity, challenging the notion of hypermasculinity as a natural or desirable state. This critique aligns with postfeminist media critiques that often expose the labor behind seemingly natural or effortless gender presentations, revealing the societal pressures that dictate strict adherence to gender norms.

The saturation of Kendom with horse symbolism and Ken's revelation about “men extenders” articulate a critique of how traditional symbols of power and masculinity are often leveraged to reinforce male dominance (00:58:33). The movie's exploration of this symbolism, culminating in Ken's realization about the performative basis of patriarchal power, provides a commentary on the mechanisms through which masculinity is asserted and maintained in society.

This portrayal not only satirizes the exaggerated aspects of male stereotypes and masculinities mentioned above but also critically examines the societal norms and expectations that perpetuate these behaviors. By presenting these traits in a hyperbolic and humorous light, the narrative invites the audience to question and reflect upon the underlying issues of gender inequality and performative character of such attitudes. The film explores Ken's development to critique the makeover paradigm and the concept of the “gaze,” noting how men, too, are objectified; it delves into gender fluidity and the effects of hegemonic and toxic masculinity.

### 2.4 Subversion of masculinity traits through uses of micro-power

The movie humorously presents the typical characteristics of masculinity within a patriarchal context, using satire to highlight these norms. When Barbie and Gloria decide to harness men's competitive nature, setting them against each other in their quest for power, they cleverly exploit these common traits and stereotypical characteristics. This strategy not only reveals the often-unspoken rules governing gendered behavior in a patriarchal society but also displays the artificiality and absurdity of such expectations. By doing so, the film deconstructs these established norms and deeply rooted performances of masculinity that have long been prevalent in various forms of representation. The narrative, therefore, becomes a tool for questioning and challenging the status quo, encouraging viewers to reconsider their own perceptions of gender and power dynamics in contemporary society.

Gloria's guidance on manipulating Kens through gendered performances prompts reflection on the tactics women employ to survive in a patriarchal society:

“Kens cannot resist a damsel in distress. You have to make them believe that you're complacent. That they have the power. And when their guard is down, you take the power back.” 01:17:31

“You have to be their mommies but not remind them of their mommy.” 01:18:19

“Any power you have must be masked under a giggle.” 01:18:22

“You can tell him that you've never seen The Godfather. And that you'd love them to explain it to you.” 01:18:41

“You have to find a way to reject men's advances without damaging their egos. Because if you say yes to them, you're a tramp, but if you say no to them, you're a prude.” 01:19:01

“Another one, be confused about money.” 01:19:12

“And then there's pretending to be terrible at every sport ever.” 01:20:03

Gloria's instructions—acting complacent, masking power with a giggle, pretending to be ignorant in sports or financial matters—highlight how women often perform prescribed gender roles to cope with the patriarchal structures that seek to define and limit their agency. This performance is a survival mechanism within a system that rewards women for conforming to subservient and stereotypically feminine roles while penalizing them for deviation (Yakalı-Çamoglu, 2017). Hence, the film goes beyond merely mocking men and their ego in hypermasculinity; it also deconstructs the conventional interpersonal dynamics between men and women within a patriarchal framework. It reveals how women have wielded micro-power in subtle ways and developed various strategies to control men and their idiosyncrasies within this system (Henley, 1973).

The humorous depiction of the Kens' eagerness to “help” the Barbies with sports serves as a critique of patriarchal courtship rituals and the broader societal expectation that men should assume a position of knowledge and authority. This scene also touches on the concept of “mansplaining,” where men feel compelled to explain things to women under the assumption that women lack knowledge or expertise. The movie critiques the constraints of performative gender roles across all genders, spotlighting both the limitations on power and agency, and the ways women resist and reclaim autonomy. This critique extends beyond merely depicting the actions of one particular gender, addressing instead the broader system and order that underpin these gender roles.

### 2.5 Doing gender, doing love

The portrayal of Ken's interactions with Barbie in Kendom, serves as a critique of toxic masculinity, illustrating how the film deconstructs such behaviors through both narrative and character development. Ken's proposition to Barbie, offering her the option to stay as his “bride wife” or “long-term-low-commitment-distance girlfriend,” alongside his later aggressive behaviors, underscores a satirical examination of toxic masculinity. These actions reflect not only a desire for dominance but also an insecurity and entitlement characteristic of toxic masculine norms. This is emblematic of the behavior observed in certain incel or misogynistic subcultures, where unreciprocated affection leads to aggressive and entitled attitudes toward women (Lindner, 2023).

The film utilizes Ken's character arc to highlight the absurdity of such toxic traits. Ken's transition from a character marked by insecurity and a desire for Barbie's approval to one who embodies aggressive dominance and entitlement when he gains power in Kendom mirrors broader societal critiques of how toxic masculinity manifests. His insistence on Barbie serving him and the symbolic act of discarding her dresses from the house represent psychological aggression and control, further illustrating the toxic dynamics at play.

This narrative strategy aligns with concept of gender performativity, suggesting that gender identities, including toxic masculine behaviors, are enacted performances shaped by societal expectations rather than innate qualities. Moreover, the movie's humorous yet critical portrayal of these dynamics engages with McRobbie's (2008) notion of the “double entanglement” of postfeminism, as it both utilizes and critiques traditional gender norms to explore complex gender relations. Barbie's response to Ken's behavior, marked by a mix of disdain and depression, reflects the emotional toll of living in a society immersed in toxic masculinity.

Another illustrative scene on the topic of gender and love is the satirical depiction of Kens playing guitars to impress Barbies on the beach. The guitar scene where multiple Kens simultaneously play their guitars and sing the same song to Barbies on the beach, creating a circle around a fire transforms the act into a repetitive, predictable, and mundane ritual, thereby serving as a symbol that exposes the performative nature of gender roles within romantic contexts. This also critiques the authenticity of such performances, suggesting they are more about conforming to societal scripts than about genuine expression.

Furthermore, this scene directly engages with the postfeminist critique of romantic narratives propagated by media and culture. Postfeminism often explores the contradictions and complexities within contemporary gender relations. The explicit acknowledgment of the act's performative nature in the dialogue between Barbie and Ken serves as a meta-commentary:

“Barbie: That's a beautiful song that you're playing. Did you write it?”

Ken- “Yes. You want to sit here and watch me do it, while I stare uncomfortably into your eyes for 4½ min?”

Barbie- “I would love that.” (01:24:35)

Inviting the audience to question the authenticity and spontaneity of gendered behaviors in courtship, the film deconstructs traditional romantic rituals by exposing their formulaic and performative aspects, thereby challenging viewers to reconsider the ways gender and love are enacted and expressed in society.

### 2.6 Men and wars

The film's fight scene, drawing inspiration from the Normandy attacks during World War II, unfolds on a beach—a setting historically associated with the utmost seriousness of warfare, combat, and sacrifice within the realm of men. However, in this movie, the concept of war and fighting is subjected to deconstruction, beginning with its underlying motivations. The Kens engage in combat not due to any external threat but as a result of psychological manipulation masterminded by the Barbies who exploit the masculine egos and competitive performativity and “petty jealousy” of the Kens. Barbies turn Kens against each other to regain power. Consequently, the Kens are portrayed as rather foolish, their egos are ridiculed, and they are intentionally depicted as comical even within the context of a seemingly serious battle.

The deconstruction of the gravity associated with war and death is exemplified through the choice of weapons. Drawing from previous fighting Barbie narratives, such as in “Barbie and the Three Musketeers,” the Kens employ unconventional items like tennis rackets, gymnastic ribbons, beach balls, and toy archers as their weaponry (Sheridan et al., 2009; Yakalı-Çamoglu, 2011). The choice of unconventional weapons and attire, such as tennis rackets and gymnastic ribbons, further diminishes the traditional gravity of war and combat, presenting these elements in a playful and absurd light.

Notably, Ken wears an Action Man attire comprising a black leather vest adorned with tasseled epaulets. Epaulets, typically worn by soldiers to signify their rank in the army, is only enjoyed by stereotypical Ken played by Ryan Gosling. His black leather trousers, along with a “Ken”-emblazoned black belt specially crafted for him and adorned with thunder strike-like figures at the bottom of the letters “K” and “N,” in addition to a black and white headband, all serve as reminiscent of Action Man in action. Ken's costume amplifies this satire by drawing on childhood symbols of masculinity.

Through a satirical deconstruction of traditional masculine ideals, especially those tied to war and combat, the film critiques and mocks stereotypical notions of masculinity. By staging a fight scene reminiscent of historical warfare on a beach, then subverting expectations with the characters' motivations and actions, it not only challenges traditional concepts of masculinity but also ties into the broader discourse of gender wars mentioned in our theory chapter. The Kens' engagement in combat, driven not by noble causes or external threats but by the Barbies' psychological manipulation of their egos and competitive nature, serves as a microcosm of the gender wars. These wars are not just literal battles but are fought on the psychological and social fronts, where masculine behaviors such as aggression, competitiveness, and the desire to assert dominance are revealed to be not innate or inherently admirable but easily manipulated and subject to ridicule.

### 2.7 Depictions of hegemonic masculinity: a critique of neo-liberal capitalist culture

In the movie, Mattel and its board serve as symbolic embodiments of both hegemonic masculinity and the actors of neoliberal capitalism. However, their portrayal is not one of intelligence but rather cunning to the point of absurdity. They are depicted as two-faced individuals who wield power; they are fully aware of the need for political correctness but show little concern for those with less power or for women.

Their headquarters is itself designed with a phallic shape and they humorously acknowledge it. The interior of the building is notably unexciting, exuding an industrial atmosphere characterized by a monotonous gray color scheme and an abundance of dreary cubicles. Within this structure, populated by exclusively male mid-management personnel, the film paints a stark picture of the “reality” of working life in a capitalist society. It becomes evident that this portrayal does not depict a contented patriarchal existence where every man finds fulfillment. Instead, the representation of the male-dominated corporate environment is dull, emphasizing the entrapment of both bodies and spirits for those who are a part of it.

The employees' monotonous and uninteresting work lives sharply contrast with Barbie's vibrant world and her colorful fashion choices. The staff members are uniformly dressed in plain canvas trousers and serious college sweaters, while the boardroom is populated by men all wearing identical black suits. Their unachieved determination to appoint women to managerial positions appears to be a response to feminist pressures. The film adopts a satirical tone when it portrays the embodiments of hegemonic masculinity during the CEO's speech, particularly when Barbie expresses surprise upon realizing the stark difference between the environment in the Mattel boardroom and her own world in Barbieland.

Barbie asks:

“Are any women in charge?”

The CEO answers in a defensive manner

“Listen, I know exactly where you're going with this, and I have to say I really resent it. We are a company literally made of women. We had a woman CEO in the 90's. And there was another one... at some other time. So that's—that's two right there. Women are the freaking foundation of this very long phallic building. We have gender-neutral bathrooms up the wazoo. Every single one of these men love women. I'm the son of a mother. I'm the mother of a son.... I'm the nephew of a woman aunt. Some of my best friends are Jewish. What I'm trying to say is... Get in the box, you Jezebel!” 00:46:37

This scene serves as a humorous critique of Mattel, shedding light on the scholarly criticisms directed toward the company. Furthermore, it acts as a reflection of the broader culture, revealing that political correctness often serves as a mere facade. In this postfeminist world, the treatment of women's roles is portrayed with a dual nature, akin to the two faces of Janus. Hegemonic masculinity remains firmly entrenched in positions of power, and feminism has only managed to make inroads into discourse, without fundamentally altering the status quo. The movie's underlying critique suggests that those in positions of power are willing to adopt subject positions in the culture if it proves profitable, with little change in their core understanding of femininity or any non-conforming subjectivity. It's crucial to recognize that the inclusion of this scene and the portrayal of Mattel in such a light constitute the core feminist critique of the Barbie Movie, as it targets the broader system rather than focusing on individuals.

## 3 Discussion

In 2016, Gill observed a transformation of feminism from a marginalized identity to a fashionable and “cool” presence within mainstream youth culture. Yet, this shift often results in an uneven focus on feminist issues in media, at times trivializing significant concerns and rendering feminist activism with limited visibility (Gill, 2016). The emergence of a neoliberal feminism, emphasizing personal empowerment over collective societal change, calls for a discerning critique. This trend commodifies feminism into a marketable yet politically detached notion in media and celebrity culture, necessitating a critical examination and challenge. Being a part and parcel of this postfeminist cultural landscape, The Barbie 2023 Movie steps into this discourse, sparking mainstream discussions on feminism, patriarchy, and notions of masculinity.

This study posits that the Barbie Movie is deeply embedded within the postfeminist media landscape. On the one hand it is the ultimate postfeminist text but on the other hand it carves out a niche for critical discussions on patriarchy, and evolving concepts of masculinity. I have suggested that the film not only commercializes feminist debates and the so-called gender wars but also leverages the longstanding academic criticism directed at Barbie to engage with these themes. Despite this commodification, the movie contributes to the discourse on masculinity, gender conflicts, and the systemic challenges influencing all genders. It not only highlights the absurdity and malleability of traditional gender performances but also points to the patriarchal structures as the narrative's true antagonist. In the realm of Kendom, Barbie is perceived as the dominating force that Kens must contend with. In the Real World, while men are at times portrayed as antagonists to confront, the presence of a postfeminist masculinity embodied by the executives of Mattell and Ken prompt us to contemplate how the film frames the patriarchal system as the antagonist in all the narratives it presents. By doing so, it underscores that the issue lies not in the gender of those in power but in the patriarchal system itself, advocating for systemic change over superficial fixes.

This analysis reveals the film's layered critique, using satire to comment on men's competitive nature within patriarchy and how women, like Barbie and Gloria, navigate and subtly subvert these norms. Barbie 2023 Movie thus becomes a reflection on postfeminist masculinity and the performative nature of gender, challenging the audience to question the authenticity of societal gender constructs. The film, with its humor and satire, deconstructs the established symbols of masculinity within patriarchy, highlighting the complex and often absurd nature of these constructs. It sheds light on the performative aspect of gender identities, emphasizing how individuals, including men, have an existential crisis within our gendered society. It highlights the notion that many of the “realities” we live by are merely facades, janus-faced socially constructed illusions.

As Ken becomes the ultimate icon of postfeminist masculinity, the film's broader critique extends to capitalism's role in commodifying social movements, including feminism, urging a deeper engagement with gender equality beyond the superficial. It also underscores the potential of popular culture as a site of resistance and critique, offering insights into the ongoing struggle for gender equality and the reimagining of masculinity in the postfeminist era. As such, the Barbie 2023 Movie is not merely a reflection of current gender discourse in the postfeminist media landscape but also opens a space for its evolution, inviting us to rethink our roles and the potential for transformative change within this landscape.

## 4 Further research

Audience reaction to the movie's gender-related themes merits further investigation in a reception research. Future studies should include how individuals of diverse gender identities interpret the movie.

## Data availability statement

The original contributions presented in the study are included in the article/supplementary material, further inquiries can be directed to the corresponding author.

## Author contributions

DY: Writing – original draft.
